# Antibiotic Resistance in Pacific Island Countries and Territories: A Systematic Scoping Review

**DOI:** 10.3390/antibiotics8010029

**Published:** 2019-03-19

**Authors:** Nicola D. Foxlee, Nicola Townell, Lachlan McIver, Colleen L. Lau

**Affiliations:** 1 Department of Global Health, Research School of Population Health, Australian National University, Canberra ACT 2600, Australia; colleen.lau@anu.edu.au; 2 Diagnostic Microbiology Development Program, Phnom Penh 12000, Cambodia; nikkitownell@hotmail.com; 3 Médecins Sans Frontières, 1202 Geneva, Switzerland; lachlan.mciver@geneva.msf.org

**Keywords:** antimicrobial resistance, gram-negative organisms, gram-positive organisms, surveillance, healthcare associated infections

## Abstract

Several studies have investigated antimicrobial resistance in low- and middle-income countries, but to date little attention has been paid to the Pacific Islands Countries and Territories (PICTs). This study aims to review the literature on antibiotic resistance (ABR) in healthcare settings in PICTs to inform further research and future policy development for the region. Following the PRISMA-ScR checklist health databases and grey literature sources were searched. Three reviewers independently screened the literature for inclusion, data was extracted using a charting tool and the results were described and synthesised. Sixty-five studies about ABR in PICTs were identified and these are primarily about New Caledonia, Fiji and Papua New Guinea. Ten PICTs contributed the remaining 21 studies and nine PICTs were not represented. The predominant gram-positive pathogen reported was community-acquired methicillin resistant *S. aureus* and the rates of resistance ranged widely (>50% to <20%). Resistance reported in gram-negative pathogens was mainly associated with healthcare-associated infections (HCAIs). Extended spectrum beta-lactamase (ESBL) producing *K. pneumoniae* isolates were reported in New Caledonia (3.4%) and Fiji (22%) and carbapenem resistant *A. baumannii (CR-ab)* isolates in the French Territories (24.8%). ABR is a problem in the PICTs, but the epidemiology requires further characterisation. Action on strengthening surveillance in PICTs needs to be prioritised so strategies to contain ABR can be fully realised.

## 1. Introduction

The prevention and treatment of infectious diseases is increasingly being challenged by the growing spread of antimicrobial resistance (AMR) [1]. AMR is responsible for an estimated half a million deaths each year in Europe and the USA and thousands more globally [2,3]. If AMR is not contained, experts estimate the excess mortality rate will rise to 10 million deaths per year by 2050 [3], with nearly half of these occurring in the Asia Pacific region [4].

In 2002, the World Health Organization (WHO) Western Pacific Region Office (WPRO) identified AMR as a public health concern for the region [5]. In 2014, countries in the region agreed to strengthen their capacity to respond to AMR, and the Action Agenda for AMR was drawn up and endorsed by member countries [6]. In 2015, WHO WPRO undertook a situational review of surveillance and health systems response to AMR in the region [7]. Their findings indicated wide variation amongst countries with respect to AMR, including the capacity to participate in regional surveillance networks; regulations around the purchase and use of antimicrobial agents; support provided by health systems in the containment of AMR; and in their understanding and awareness of AMR [6,7].

Pacific Islands Countries and Territories (PICTs) are amongst WHO WPRO’s low- and lower-middle-income groups, except for PNG and Tuvalu which are considered middle-income [8]. PICTs face unique challenges when it comes to addressing AMR. Their small size, remoteness, limited resource bases, fragile health infrastructures and susceptibility to natural disasters make them particularly vulnerable to AMR [7].

Several research studies have investigated AMR in low and middle-income countries [1,9], but to date, little attention has been paid to PICTs. Many PICTS have limited clinical diagnostic and laboratory capacity, which affects the availability and quality of culture and susceptibility testing data. Therefore, in most PICTs the resources and capacity to conduct good quality research into AMR is limited and published evidence on AMR in the region is scarce [10]. A scoping study was undertaken to map the evidence about antibiotic resistance (ABR) in PICTs to gain an overall understanding of ABR in the region. The findings may also inform future policy initiatives and strengthen health system adaptive capacity to contain AMR.

A scoping study was chosen for two reasons: because the literature about ABR for this region is limited and because the scoping study design allows for the capture of a broad range of results regardless of study design.

## 2. Methods

### 2.1. Geographic Setting

The PICTs included in this scoping study are the 22 low- and middle-income country members of the Secretariat of the Pacific Community (http://www.spc.int/): Cook Islands, Federated States of Micronesia (FSM), Fiji, French Polynesia, Guam, Kiribati, Marshall Islands, Nauru, New Caledonia, Niue, Northern Mariana Islands, Palau, Pitcairn Islands, Papua New Guinea (PNG), Samoa, American Samoa, Solomon Islands, Tokelau, Tonga, Tuvalu, Vanuatu, Wallis and Futuna.

### 2.2. Search Strategy

Literature searches were conducted using the following databases: PubMed, Embase, SCOPUS and Web of Knowledge accessed through the Australian National University Library system. The online systems of the National Library of Australia and the WHO Western Pacific Region (WPR) International Research Information Service (IRIS), the WPR Index Medicus (WPRIM) and Google Scholar were searched for additional articles and grey literature. Select key terms used included *antimicrobial resistance, antibiotic resistance, antibacterial agents* or *drug* or *multi-drug* combined with *resistance or susceptibility*. These terms were combined with the associated database descriptors and searched across each named PICT. The reference lists of articles and reports retrieved were searched manually for additional citations. All retrieved items were entered in an Endnote library. The details of the databases accessed, and the final search strategies used for two databases can be found in Supplementary Material S1. (Databases and grey literature sources accessed and search strategies for two databases).

Inclusion criteria:

1. Reports on ABR in humans in PICTs;
2. Published in English or French between 1950 and 2018;
3. Available in full text.

Exclusion criteria

1. Reports about tuberculosis; (in view of solid literature base already known about drug resistant tuberculosis, and globally funded TB program);
2. Literature which did not provide details about antibiotic susceptibility in PICTs;
3. Conference abstracts and posters; and newspaper articles.

### 2.3. Selection and Screening

**Figure 1:**
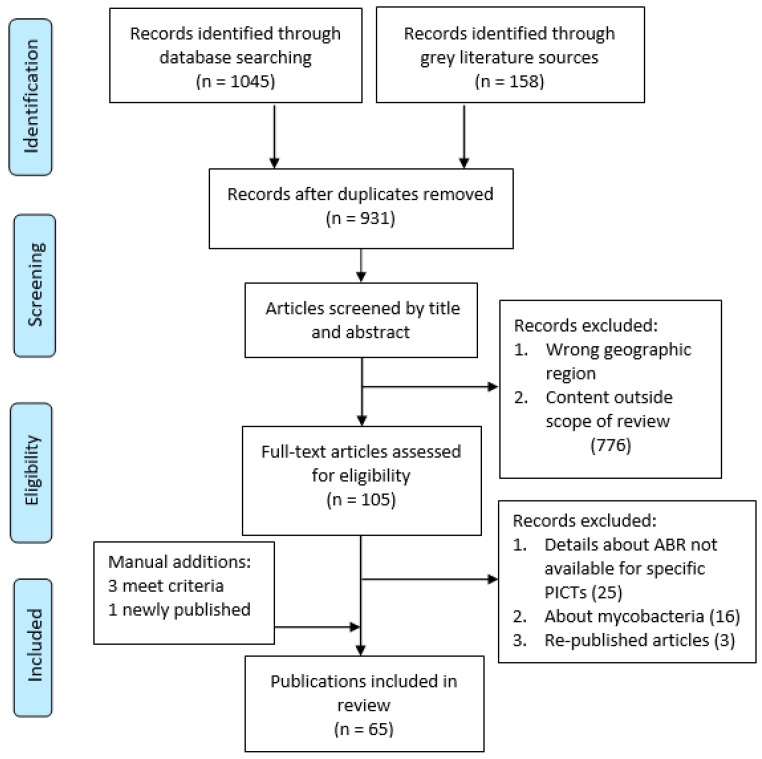
Prisma flow chart detailing article selection process.

The titles and abstracts of all results retrieved underwent an initial screen guided by minimum inclusion and exclusion criteria by one member of the team. Duplicates and candidate studies not meeting the criteria were excluded. The full text of all remaining studies was obtained and stored in the Endnote library. Four members of the team working independently carried out a second screening of the studies using the full inclusion and exclusion criteria. In situations where there was uncertainty, the team members reached a decision through discussion. If published reviews included data that were available from original articles, the data were extracted from the original articles. A flow chart (Figure 1) provides details of the number of items screened and assessed for eligibility.

### 2.4. Data Extraction

A charting tool in the form of an Excel spreadsheet was developed by two members of the team to extract and record the following information about each article: first author; publication date; country of focus; date research conducted; sample size; study design; age group; type of infection; bacteria isolated; specimen types; antibiotic susceptibility testing method; and antibiotics tested; acquisition being community or healthcare associated. Publications were broadly categorised under the following types: journal articles and published reports. Journal articles were further classified into randomised control trial, clinical trial, prospective and retrospective cohort, case control study, cross sectional survey, case study, descriptive study, laboratory study and antibiotic guideline. Reports included surveillance and outbreak reports. Guidelines included antibiotic guidelines which included antibiograms. Age groups were categorised as neonate (<1 year ), infant (≥1 to <3 years), children (3 to ≤18 years), and adult (>18 years). The results of the charting process are detailed in Supplementary Material S2. (Characteristics describing studies reporting gram-negative and gram-positive pathogens in PICTs), which describe the studies reporting gram-negative bacteria and gram-positive bacteria respectively.

### 2.5. Synthesis of Results

The studies were divided according to whether the bacterial pathogens analysed are gram-negative or gram-positive. Within these two broad categories, the findings reported about each bacterium, including the PICT(s) in which it was reported, the infections the bacterium caused, the population involved, and the level of susceptibility reported for each antibiotic tested is summarised. Gram-negative bacteria causing healthcare associated infections are grouped together and summarised before other gram-negative bacteria. The summary is supported by seven tables, which describe the study characteristics and report the prevalence of ABR in selected organisms by PICT and study date. Further details can be found on the PRISMA-ScR checklist [11] (Supplementary Material S3: Preferred Reporting Items for Systematic reviews and Meta-Analyses extension for Scoping Reviews (PRISMA-ScR) Checklist).

## 3. Results

### 3.1. Study Characteristics

Sixty-five studies, reports and guidelines met the selection criteria and provided information about ABR in 12 of the 22 PICTs. The publications were primarily about PNG (*n* = 26), Fiji (*n* = 8) and New Caledonia (*n* = 10). Four studies were conducted in French Polynesia, two each in Cook Islands and Samoa and one each for Solomon Islands and Wallis and Futuna. Fiji, Kiribati, Marshall Islands, Micronesia, PNG, Samoa, Solomon Islands and Tonga were all mentioned in the eight studies which included multiple PICTs. Three studies reference ABR in Pacific Islanders living in countries outside of the PICTs. Most (48%) studies focused on community-acquired infections, while 16% focused on healthcare-acquired infections (HCAIs) and 17% on both. The source of acquisition was unclear in the remainder (19%). Further details can be found in Supplementary Material S2 which provide details of the studies referring to gram-negative and gram-positive bacteria, respectively.

### 3.2. Antibiotic Susceptibility Test Methods

Several methods were used to determine resistance patterns. The most widely used were the disk diffusion and E-test gradient diffusion methods. Disk diffusion was used alone in 11 (17%) studies and combined with other methods in 26 (40%), whilst E-test gradient diffusion was combined with disk diffusion in 18 studies and used alone in two (3%). The agar dilution and broth microdilution techniques, the replica plating method, the automated VITEK system, the ATB PNO strip and molecular detection (PCR) were all used as the sole method in seven (11%) studies or in combination with another method. The remaining studies did not report the method used (19, 29%).

### 3.3. ABR Reported in PICTs in This Scoping Study

#### 3.3.1. ABR in Gram-Negative Bacteria: Healthcare-Associated Infections (HCAIs)

##### *Klebsiella* spp.

Studies about ABR resistance in *Klebsiella* spp. were identified for PNG, New Caledonia, Fiji, Kiribati and FSM [12,13,14,15,16,17,18,19,20,21]. Table 1 provides additional details. In 1987, a review of ABR in the PNG Highlands found > 50% of *Klebsiella* spp. (*n* = 22) to be multidrug-resistant (MDR) to chloramphenicol 45%; ampicillin 95%; tetracycline 36% and cotrimoxazole 32%. The isolates exhibited susceptibility to gentamicin [18]. In contrast, a single case of MDR *K. oxytoca* was reported in 1992 in Port Moresby General Hospital (PMGH) [20]. The isolates were resistant to gentamicin, streptomycin, chloramphenicol and cotrimoxazole, but susceptible to tetracycline. Gentamicin resistance was observed in *Klebsiella* spp. isolated from blood cultures from two patients at PMGH in 1991–2 [20]. An outbreak of *K. pneumoniae* bloodstream infections occurred during 2007 and 2008 in the special care nursery in PMGH (*n* = 57) [16]. In 2007, 16 (74%) isolates were cephalosporin-sensitive and four (25%) were multidrug-resistant (*n* = 20), while in 2008, 10 (27%) were cephalosporin-sensitive, 21 (57%) multi-resistant and six (16%) resistant to all antibiotics (the specific antibiotics were not reported) (*n* = 37) [16]. In PNG in 2012, resistance to third generation cephalosporins was reported to be 63.5% (*n* = 252) [21].

At the Centre Hospitalier Territorial, New Caledonia between 2008 and 2013, 18% of patients with bacteraemia had infection with community-acquired *K. pneumoniae* (*n* = 119) [17]. Fifty-four percent (64/119) of isolates showed susceptibility to several classes of antibiotic, including amoxicillin clavulanic acid, quinolones, cephalosporins, macrolides, aminoglycosides and co-trimoxazole. Extended spectrum beta-lactamase (ESBL) production, determined using the double disk potentiation method, was found in four isolates 3.4% (4/119). In contrast, *K. pneumoniae* reported to be ESBL-producing was isolated from 22% (94/437) of bacterially confirmed HCAI in the adult intensive care unit (ICU) in Fiji’s Colonial War Memorial Hospital (CWMH), during 2011 and 2012 [19]. In the same hospital during 2012, *K. pneumoniae* represented 20% (21/103) of organisms isolated from neonatal ICU patients with suspected sepsis. Results for the combined gram-negative organisms isolated indicated antibiotic resistance to gentamicin 45%, ceftriaxone 48% and ciprofloxacin 25% [15]. According to a 2014 WHO surveillance report based on 20011/12 data, resistance of *K. pneumoniae* isolates to third generation cephalosporins in Kiribati was 1% (*n* = 111), Fiji 25% (*n* = 2900), FSM 71% (*n* = 87) and 63.5% (*n* = 252) in PNG. Only one report of carbapenem resistance was identified and this was in 0.7% of isolates from two hospitals in Fiji (*n* = 2175) [21].

##### *Acinetobacter* *Baumannii*

*A. baumannii* was reported in studies about HCAIs in French Polynesia, New Caledonia and Fiji [15,19,22,23,24]. Carbapenem-resistant *A. baumannii* (CR-*Ab*) was isolated from clinical specimens in 2004 from hospitals in both French Polynesia and New Caledonia [22,23,24]. Twenty-four patients in French Polynesia were either colonised (19; 80%) or infected (5; 20%) with *A. baumannii*. This bacterium was linked to pneumonia, skin and soft tissue, surgical site or blood stream infections. The isolates were resistant to all beta-lactams and 20 (83% 20/24) were susceptible to colistin and aminoglycosides [24]. In New Caledonia, CR-*Ab* isolates represented 24.8% (123/202) of multidrug- resistant bacteria (*n* = 202); the isolates were resistant to beta-lactams, quinolones and aminoglycosides except amikacin and tobramycin, but susceptible to colistin [22,23]. The types of infections in French Polynesia were like those found in New Caledonia, but also included urinary tract infections. Both outbreaks were associated with the OXA-23 producing *A. baumannii* clone [22,23,24]. HCAIs were the subject of two studies conducted in Fiji at CWMH during 2011 and 2012 [15,19]. These studies involved patients in adult and neonatal ICUs. Neither study provided antibiotic susceptibility data. The adult ICU study found that 21% (92/437) of isolates cultured from blood, respiratory tract, surgical site and urinary specimens to be *Acinetobacter* spp. [19]. In the neonatal ICU study, *A. baumannii* was responsible for 14.5% (15/103) of gram-negative sepsis [15].

##### *Pseudomonas* *aeruginosa*

*Pseudomonas aeruginosa* was reported in PNG, New Caledonia, Fiji, Samoa and Cook Islands [12,14,18,19,23,25,26]. In PNG in 1999, 11 *P. aeruginosa* isolates were cultured from blood specimens taken from 54 children with severe sepsis in Goroka Hospital and 82% were resistant to gentamicin [1]. In 2009 at the Modilon Hospital in PNG, one HCA *P. aeruginosa* isolate (1/9; 11%) was found to be resistant to tetracycline, cotrimoxazole, chloramphenicol and ampicillin, but susceptible to ciprofloxacin and gentamicin [12]. A laboratory study in 2004 in New Caledonia found seven (3.5%) ceftazidime-resistant *P. aeruginosa* isolates amongst other MDR bacteria (*n* = 202) [23]. Isolates collected from all sources in 2015 and 2016 in Samoa were 24% (9/37) resistant to ciprofloxacin. Reduced susceptibility was shown to gentamicin [26]. *P. aeruginosa* isolates cultured from all sources between 2015 and 2017 at the Rarotonga Hospital microbiology laboratory, Cook Islands were shown to have reduced susceptibility to ceftazidime (*n* = 117) and, ciprofloxacin and gentamicin (*n* = 154), but all were susceptible to meropenem [25].

##### *Enterobacter* spp.

Four studies reported on *Enterobacter* spp. for PNG, Fiji and French Polynesia [14,19,27,28]. A 1997 investigation into severe sepsis in children in Goroka Hospital, PNG found 12% (7/61) of positive cultures to be *Enterobacter* spp.: three were HCAIs and four were community-acquired [14]. Whilst all were resistant to chloramphenicol, four were resistant to gentamicin. In 2007 an outbreak investigation of *E. aerogenes* in the neonatal ICU in Fiji’s CWMH found 55.5% (10/18) of septicaemia were caused by ESBL producing *E. aerogenes* [28]. The isolates were resistant to ampicillin, trimethoprim-sulfamethoxazole, gentamicin, cephalothin, and ceftriaxone, but remained susceptible to meropenem, amikacin with intermediate resistance to ciprofloxacin. In 2015 a single isolate of imipenem-resistant (IMI-1 producing) *E cloacae* was detected in an adult male in the main hospital in Papeete, French Polynesia [27]. This isolate was resistant to aminopenicillins and carboxypenicillins, amoxicillin/clavulanic acid, first and second generation cephalosporins, as well as imipenem. The isolate remained susceptible to the ureidopenicillins, to ceftazidime, cefepime and cefotaxime, meropenem, ertapenem and doripenem, as well as the non-beta-lactams [27].

#### 3.3.2. ABR in Gram-Negative Bacteria: Community-Acquired Infections

##### *Neisseria* *gonorrhoeae*

Sensitivity and resistance patterns for *N. gonorrhoeae* were reported for New Caledonia, Fiji, PNG and Solomon Islands [29,30,31,32,33,34,35,36,37,38] (Table 2). According to the WHO Gonococcal Antimicrobial Surveillance Program (GASP) [29,30,31,32,35], there has been widespread resistance to penicillin in the Western Pacific for many years (Table 2). In 2011 resistance to ciprofloxacin and penicillin in New Caledonia was estimated to be 6% (10/166) and 9% (15/166) respectively [29,35]. While resistance to penicillin in Fiji was reported to be 6.7% (17/252) [29,35], the rate in PNG ranged from 40% to 63% (21/52; 34/64)) at this time [29,30]. Resistance to quinolones in 2010 in PICTs was low (Fiji 0.6% (2/366) New Caledonia 0.5% (1/197)) [31]. In 2012 reduced susceptibility to third generation cephalosporin was reported in Fiji and Solomon Islands at 0.4% (*n* = 541) and one in ten isolates, respectively [21].

##### *Escherichia* *coli*

Several studies reported ABR in *E. coli* isolates in PNG, Fiji, FSM, Kiribati and Samoa (Table 3) [14,15,18,21,26,39,40]. The earliest study was in PNG in 1987. In this study the rate of *E. coli* resistance was 32% (12/37) to chloramphenicol, 46% (17/37) to ampicillin and 3% (3/37) to gentamicin [18]. Ten years later (1997) this resistance to chloramphenicol and gentamicin had risen to 96% (59/61) and 37% (22/61) respectively [14]. A WHO surveillance study (2002) [40] reported the rate of resistance of *E. coli* in Fiji was 50% for ampicillin and 10% for gentamicin, while resistance to gentamicin in Tonga was <5%. The number of isolates and specimen types were not reported [40]. In 2012 in Fiji, 24% of *E. coli* isolates (*n* = 103) were found to have high rates of resistance to ampicillin (87%; 64/74) and gentamicin (45%; 34/77) [15]. Resistance to third generation cephalosporins in 2012 was 12.2% (*n* = 2895) for Fiji, 77% (*n* = 158) for FSM, 24.1% (*n* = 174) for PNG and 12.9% (*n* = 43) for Samoa [21], while resistance to the fluoroquinolones was 11.9% (*n* = 2566) for Fiji, 3% (*n* = 72) for Kiribati, 16% (*n* = 158) for FSM, 13.3% (*n* = 526) for PNG, and 13.9% (*n* = 43) for Samoa [21]. There were no reports of carbapenem-resistant *E. coli*.

##### *Salmonella* spp.

Seven studies about resistance patterns in *Salmonella* spp. in PICTs were identified (Table 3) [18,21,40,41,42,43,44]. The earliest study (1987) about non-typhoidal *Salmonella* (NTS) was from PNG’s Eastern Highlands Province [18], where 58% (22/38) of isolates were resistant to chloramphenicol, 37% (14/38) to kanamycin, 53% (20/38) to ampicillin and 6% (2/38) to tetracycline. In 2012 NTS resistance to fluoroquinolones in PNG was 33.3% (5/15) [21]. In contrast, although *S. typhi* has been reported increasingly in Fiji since 2005, ABR was uncommon [44]. Ninety-six *S. enteritidis* isolates cultured during an outbreak in French Polynesia (2008 to 2013) were the subject of a laboratory study [43] and resistance to amoxicillin was found in only three isolates. A surveillance study conducted in 2011 in the PNG Highlands following a cholera outbreak found 2.3% of isolates to be *S. typhi* (5/216) [41]: all were resistant to ampicillin, tetracycline, co-trimoxazole and chloramphenicol and one isolate was also resistant to nalidixic acid. In Samoa between 2015 and 2016, *S. typhi* isolates from blood samples exhibited reduced susceptibility to amoxicillin and ampicillin (*n* = 22), ceftriaxone (*n* = 50), ciprofloxacin (*n* = 56) and gentamycin (*n* = 58) [26]. In New Zealand, there are higher rates of *S. typhi* in Pacific Islanders than in other ethnic groups [42]. Samoans and Tongans comprise ~7.4% of the New Zealand population, but account for >30% of locally acquired cases [42]. In 2012 there were 41 cases of *S. typhi* reported, 50% were resistant to nalidixic acid and 30% of patients were of Samoan ethnicity [42]. Table 3 provides additional details about studies in this pathogen.

##### *Shigella* spp.

Five studies reported on *Shigella* spp. in PICTs (Table 3) where *S. flexneri* is the predominant serotype [18,26,40,41,45,46,47]. In 1987 in PNG *S. flexneri* isolates (78/94) were resistant to chloramphenicol (83%), tetracycline (91%), and ampicillin (86%), but susceptible to gentamicin and cotrimoxazole [18]. Four percent of isolates cultured from stool samples collected between 2000 and 2009 in PNG were also found to be *S. flexneri*. (136/3419) [45,46]. These isolates were resistant to amoxicillin (96%; 131/136), chloramphenicol (60%; 82/136), and co-trimoxazole (86%; 117/136), but susceptible to ciprofloxacin and cephalexin. In 2014, again in PNG, 21.8% (47/216) of isolates also from stool samples were *Shigella* spp. [41]. The resistance rates from these isolates were similar to findings from earlier studies: ampicillin (91.5%), tetracycline (76.6%), co-trimoxazole (70.2%) and chloramphenicol (55.3%). None of the isolates were resistant to ceftriaxone or ciprofloxacin, but 55.3% (26/47) were multidrug-resistant to up to four antibiotics [41]. Fiji reported 575 cases of *Shigella* spp. between 1996 and 1998, including four deaths [47]: 30% (169/575) were reported by Lautoka Hospital and 75% (127/169) were *S. flexneri*. The *S. flexneri* isolates were >80% resistant to ampicillin and chloramphenicol, 77% resistant to doxycycline and 30% to trimethoprim [47]. In 2002 according to national data sources [40], *S*. *flexneri* isolates in Fiji were tri-resistant to ampicillin, chloramphenicol and tetracycline, but susceptible to co-trimoxazole. In contrast, *S. flexneri* isolates from Tonga were 90% resistant to both ampicillin and co-trimoxazole, but susceptible to tetracycline and chloramphenicol [40].

##### *Vibrio* *cholerae*

A cholera outbreak was reported in PNG between 2009 and 2012 in Morobe Province [48]. Between 2009 and 2011, *V. cholerae* was isolated from 95% (305/321) of stool and rectal samples. The isolates were resistant to amoxycillin (75.8%), erythromycin (38.2%), co-trimoxazole (3.2%) and tetracycline (9.7%), but susceptible to norfloxacin, ciprofloxacin and nalidixic acid.

##### *Campylobacter* spp.

Antibiotic resistance in *Campylobacter* spp. was reported for PNG [18]. The bacteria were isolated from blood, stool, lung and skin tissue, and urine samples between 1984 and 1986: 22 (*n* = 22) isolates of *C. jejuni* and 33 of *C. coli* were resistant to cotrimoxazole (100%) and ampicillin (24%), but remained susceptible to chloramphenicol, tetracycline and gentamicin [18].

### 3.4. ABR in Gram-Positive Bacteria

#### 3.4.1. *Staphylococcus aureus*

*S. aureus* were identified in 18 studies about PICTs and in reference to Pacific Islanders living abroad [12,21,25,26,49,50,51,52,53,54,55,56,57,58] (Table 4). The earliest study was from the Cook Islands in 1959 [57], where 77% of isolates (150/195) cultured from infected skin lesions collected in Rarotonga were *S. aureus*. Penicillin resistance was found in 24% (26/150) of the isolates and six isolates were multidrug-resistant to a range of antibiotics (not listed). The first report of community-acquired methicillin resistant *S. aureus* (nmMRSA) in PICTs was in 1983 in PNG [58]. In this study 98% (391/399) of isolates from infected skin sores in children were resistant to penicillin (all β-lactamase producing) and three isolates were also resistant to methicillin and multidrug-resistant to erythromycin, chloramphenicol and tetracycline [58]. Healthcare-associated MRSA was first reported in Goroka Base Hospital in 1987 (18): 97% (71/73) of isolates were resistant to penicillin, 3% (2/73) to methicillin, 1% (1/73) to erythromycin, 7% (5/73) to chloramphenicol and 3% to tetracycline. A 2012 study investigating bloodstream infections in surgical patients in Modilon Hospital, PNG found *S. aureus* in 3.5% (4/115) of isolates, of which three were MRSA [12]. The MRSA isolates remained susceptible to chloramphenicol [12]. According to National data collected in PNG in 2012, 43.9% of *S. aureus* isolates cultured from blood, urine and wound specimens were MRSA (72/164) [21]. A 2018 study investigating MRSA in children with osteomyelitis from PNG found 67% of isolates to be *S. aureus* (47/70) and predominantly nmMRSA [49]. The resistance to antibiotics tested included: penicillin 91.5%, methicillin 85.1%, oxacillin 89.4%, ampicillin 93.6%, and ceftriaxone 80.9% [49].

Two studies reported MRSA in Samoa and Fiji in 2011 and 2014, respectively. The Samoan study conducted in 2007–2008 isolated *S. aureus* from 47% of wound swabs (187/399) taken from both inpatients and outpatients [50]: 82% (153/187) were methicillin-susceptible (MSSA), whilst 18% (34/187) were nmMRSA. Sixty-eight percent of the MRSA isolates were resistant to only β-lactams and 36% were resistant to either both ciprofloxacin and erythromycin (24%) or one of the antibiotics (11%). The MSSA isolates were only resistant to penicillin (38%) [50]. The 2014 study from Fiji reported on two research projects conducted between 2006 and 2007: one on community-acquired *S. aureus* (*n* = 455) and the other on MRSA (*n* = 36) in a hospital setting [53]. While 57.4% (261/455) of the isolates from impetigo lesions in children were found to be *S. aureus*, the prevalence of nmMRSA among them was 6.7% [53]. The hospital study found multidrug-resistant MRSA in 39% (14/36) of isolates and these were recognised as mMRSA. The remaining 61% were nmMRSA and likely to be community-acquired [53]. In 2012 the prevalence of MRSA ranged from 31% (*n* = 36) in Kiribati, 24% in Samoa (*n* = 389), 17.2% (*n* = 430) in Tonga and 4% (*n* = 113) in FSM as reported by national sources [21]. In 2016 *S. aureus* in Samoa were found to be 42% (179/428) resistant to flucloxacillin, 33% (312) to erythromycin, 47% (216) to chloramphenicol, 20% (56/287) to clindamycin and 10% to doxycycline (35/318) [26]. Isolates from Cook Islands in 2017 were 90% (495/550) resistant to amoxicillin, ampicillin and penicillin; 10% to clindamycin (33/327); 22% (129/588) to flucloxacillin and 17% (94/557) erythromycin. The MRSA rate in the Cook Islands appears to have remained stable at ~11% during 2012 to 2017 [25].

Several studies focused on *S. aureus* in Pacific Islanders living in Hawaii [56], New Zealand [59] or Australia [52]. The findings indicated that patients from PICTs were over-represented among cases with nmMRSA. In Hawaii, where Pacific Islanders comprise 24% of the total population, 51% of nmMRSA isolates (176/346) were cultured from specimens taken from Pacific Islanders [56].

#### 3.4.2. *Streptococcus pneumoniae* and *Haemophilus influenzae* (Respiratory Tract Infections)

Patterns of antibiotic resistance in *Streptococcus pneumoniae* were reported in several studies about PNG, New Caledonia, Wallis and Futuna, and French Polynesia [57,60,61,62,63,64,65,66,67,68,69,70,71,72,73,74,75,76] (Table 5). *S. pneumoniae* and *H. influenzae* type b (Hib) are reported under the same heading in this study, as both bacteria cause diseases of the respiratory tract and tend to be detected together [77]. ABR in both *S. pneumoniae* and *H. influenzae* type b (Hib) are reported in six studies from PNG [61,62,63,65,66,67,72].

The first report of penicillin-resistance in pneumococci was in PNG in 1969 [73,74]. The prevalence of penicillin-resistant pneumococci isolates (*n* = 503) collected from both carriers and patients with pneumonia and meningitis was 12% (64/503). The isolates were sensitive to ampicillin, tetracycline, chloramphenicol, erythromycin, lincomycin and sulphadiazine-trimethoprim. Resistance to penicillin in pneumococci isolates in PNG increased to 14% (41/292) between 1971 and 1974 [64]. Findings indicated that the resistant isolates were three times more common amongst carriers (18%; 34/191) than patients with pneumonia or meningitis (6%; 6/101). By 1978 resistance to penicillin had increased to 33% (19/57) in patients with bacteraemic pneumonia and meningitis in PMGH [64]. In 2008, also in PMGH 88% (45/51) of bacteria isolated from CSF specimens were pneumococci [75]. Whilst these pneumococci isolates were susceptible to erythromycin, cefaclor, ceftriaxone and ampicillin, they exhibited reduced susceptibility to tetracycline (27/28), penicillin (37/40), and chloramphenicol (35/38) [75].

Two retrospective studies investigated ABR in invasive *S. pneumoniae* and *H. influenzae* (Hib) isolates in children in PNG, from 2006 to 2009 in Modilon Hospital and 1996 to 2005 in Goroka Hospital [65,76]. In both studies isolates from blood and/or CSF specimens were taken from hospitalised patients prior to the 2008 and 2014 roll-out of the Hib and PCV13 vaccines, respectively. The Modilon study found all Hib isolates (*n* = 15) were chloramphenicol-resistant, while *S. pneumoniae* isolates (*n* = 17) exhibited reduced susceptibility. All isolates were susceptible to ceftriaxone [76]. The Goroka study conducted between 1996 and 2005 revealed ABR in both pathogens was demonstrated across most antibiotics tested during the period [65]. Some 32.5% (53/165) of *H. influenzae* isolates type B (Hib) were beta-lactamase-positive and resistant to ampicillin and cotrimoxazole. In addition, 98% (161/165) and 96% (158/165) of the Hib isolates were resistant to chloramphenicol and tetracycline respectively. A total 21.5% (39/180) of *S. pneumoniae* isolates were penicillin-resistant and 4% were either cotrimoxazole, tetracycline or chloramphenicol resistant (7/180) [65]. To determine any increase in ABR over time, two periods were compared: 1996–2000 and 2001–2005. Results indicated there was no statistical difference in the proportions of *S pneumoniae* isolates that were resistant pre- and post-2000, but the proportions of *H. influenzae* isolates resistant to ampicillin and chloramphenicol increased from 26% (27/104) to 46.6% (27/58) and 26% (27/104) to 41.4% (24/58), respectively.

The serotypes responsible for penicillin-resistance and associated with *S. pneumoniae* were examined in a study from New Caledonia in 2005 (*n* = 298) [70]. Invasive isolates were cultured from blood (37%), spinal fluid (6%) and respiratory specimens (41%). Findings indicate that 14.4% (43/298) of isolates had reduced susceptibility to penicillin, with five isolates being fully resistant. Some 3.7% (12/298) expressed intermediate susceptibility to amoxicillin and 1.7% (5/298) to both amoxicillin and cefotaxime. Several isolates belonged to the group of serotypes present in the Pacific, which are more likely to express reduced susceptibility to penicillin [70].

Two nasopharyngeal carriage studies in infants < 2 years reported on antibiotic resistance in *S. pneumoniae* in New Caledonia and Fiji in 2005 and 2006, respectively [60,71]. In New Caledonia, 52% (544/1040) of nasopharyngeal specimens were positive for *S. pneumoniae* and 21% (114/544) were penicillin-resistant [60]. Although, penicillin-resistance in the Fiji study was lower at 11.4% (28/246), resistance to cotrimoxazole was 20.3% (50/246). Approximately 72% (20/28) of isolates resistant to penicillin were also resistant to cotrimoxazole. Two isolates were also resistant to ceftriaxone, four to erythromycin and three isolates were multidrug-resistant. All isolates were fully susceptible to chloramphenicol [71]. The serotypes with reduced susceptibility in both Fiji and New Caledonia included those identified in the earlier 2005 New Caledonian study mentioned above [60,70,71].

#### 3.4.3. *Streptococcus pyogenes (Group A Streptococcus)*

Only four studies reported on susceptibility patterns in *Group A Streptococcus*: PNG, New Caledonia, Fiji and Samoa [50,58,78,79]. Ninety (90) cases of *Group A Streptococcus* were recorded in New Caledonia during 2006. Isolates from skin and soft tissue, blood and, pleural, spinal and amniotic fluid were 10% resistant to tetracycline, but susceptible to all other antibiotics tested including; penicillin, amoxicillin, gentamycin, erythromycin, vancomycin, streptomycin and rifampin [78]. In 2016 in Samoa, 15% of *Group A Streptococcus* isolates (11/78) were resistant to erythromycin and 23% to (53/70) and clindamycin [50].

##### *Enterococcus* spp.

Two studies examining *E. faecalis* and *E. faecium* were identified [80,81]. A single case of vancomycin resistant *E. faecalis* in a patient hospitalised for an intestinal obstruction was reported in New Caledonia in 2006. Three isolates cultured from stool and urine samples were resistant to vancomycin and teicoplanin due to the presence of the *vanA* gene, as well as to streptomycin, erythromycin and kanamycin [81].

## 4. Discussion

Sixty-five studies on ABR in PICTs spanning five decades (1958–2018) were identified. Whilst the available data suggest widespread ABR across PICTs, there was scarce or a complete absence of data from several countries.

*S. aureus* was the predominant gram-positive pathogen, mainly in association with skin and soft tissue infections in the community setting. Rates of MRSA ranged widely: ≥50% in PNG and > 20% in other PICTs [25].

Several gram-negative bacteria listed on the WHO Priority Pathogen List [69] were reported in PICTs, including *A. baumannii*, *P. aeruginosa K. pneumoniae* and *E. coli.* All were reported in PICTs in association with HCAIs including pneumonia, sepsis, catheter-related bloodstream, surgical site infections and UTIs. Significant rates of resistance in *K. pneumoniae* (>50%) and *E. coli* (12% to 50%) to third generation cephalosporins were reported in several PICTs [15,21]. Reports about confirmed ESBL-producing organisms were limited to *K. pneumoniae* and *E. aerogenes* in New Caledonia and Fiji [17,19,28] and carbapenem resistance *in A. baumannii* isolates in the French Territories: New Caledonia and French Polynesia [23,24]. Whilst other gram-negative pathogens were reported in PICTs including *P. aeruginosa* and *S. typhi* [40], the studies were few and the antibiotic susceptibility patterns reported varied widely.

These findings are particularly concerning as antibiotic options, to treat life threatening infections caused by multidrug-resistant organisms, are limited in PICTs due to cost and availability.

Our findings suggest that for severe gram-negative HCAIs, alternative broad-spectrum therapies, such as the carbapenems will likely see an increase in use. However, if the emerging resistance to the carbapenems continues [22,23,24], last line drugs such as colistin will be required. Health systems in PICTs will find it difficult to fund these last line antibiotics, which are often more expensive, toxic and not readily available.

Whilst this scoping study confirms the presence of MDR organisms in the region, interpretation of the literature and application of the data is challenging for several reasons. Firstly, the results represent only 54.5% (12) of the 22 countries included in the review. Most studies (72%) are about PNG, Fiji and New Caledonia. Eighteen percent (18%) refer to multiple PICTs and 10% are about individual PICTs. Secondly, this is a scoping study and no attempt has been made to judge the quality of the data. Microbiology laboratories in majority of PICTs were restricted to larger urban areas and results may not be representative of the whole country. The methodologies and breakpoints used for testing antibiotic susceptibility varied across PICTs and were not consistently reported. Performing microbiology testing in PICTs is challenged by a shortage of trained staff, consumables and equipment and this limits the availability of quality diagnostic testing. Storing, transporting and organism identification also present difficulties [82]. Thirdly, several studies have limited data, which increases the possibility of results being biased and ABR being over- or underestimated.

## 5. Conclusions

The epidemiology of antimicrobial resistance in the Pacific requires further characterisation. Available data were limited to a few major centres and further studies are needed to provide greater representation of the region. Our study found that data were too heterogenous for comparisons within and between countries. Therefore, it is imperative that future studies capture local data that allow comparison between settings, including hospital verses community infections, and urban, rural and outer island populations.

Improved surveillance data from PICTs to support efforts to contain AMR at the local, national and international levels are urgently needed. At the local level, surveillance data can be used to inform infection control programs and develop locally relevant antibiotic guidelines. Although several PICTs have published their own guidelines, much work remains to be done to produce guidelines for specific infectious diseases; and to ensure recommendations remain current with local microbiology and susceptibility patterns.

The literature supports the need for a formal introduction of antimicrobial stewardship programs in PICTs, which include developing and disseminating these guidelines; improving awareness of AMR; improving antibiotic prescribing behaviours; and implementing infection prevention and control practices. This will contribute to containing AMR in PICTs.

## Figures and Tables

**Table 1:** Prevalence of ABR in *Klebsiella pneumoniae* and *Escherichia coli* in PICTs reported by country and date.

***K. pneumoniae***				
**Antibiotic Class**	**Antibiotics**	**PICTs**	**1980–2000** **% or % Range (No. Isolates Tested per Study)**	**2011–2017** **% or % Range [No. Isolates Tested per Study]**
Cephalosporin	Ceftriaxone Cefotaxime Ceftazidime	Fiji Micronesia PNG		25 (2900) 71 (87) 63.5 (252)
	Ceftriaxone	Samoa		7.7–19.8 (116, 119)
Carbapenem	Imipenem Meropenem	Fiji		0.7 (2175)
Aminoglycoside	Gentamicin	PNG	100–78.4 (14, 41)	
Tetracycline	Tetracycline	PNG	36 (11)	
Chloramphenicol	Chloramphenicol	PNG	40–100 (14, 22)	
	Nalidixic acid	New Caledonia		54 (119)
Fluoroquinolone	Ciprofloxacin	New Caledonia		54 (119)
Diaminopyrimidine	Co-trimoxazole	New Caledonia		54 (119)
		PNG	32 (22)	
***E. coli***				
**Antibiotic Class**	**Antibiotics**	**PICTS**	**1980–2000** **% or % Range (No. Isolates Tested per Study)**	**2011–2017** **% or % Range (No. Isolates Tested per Study)**
	Ampicillin	PNG Fiji	46 (37)	87 (25)
Cephalosporin	Ceftriaxone Ceftazidime Cefotaxime	Fiji Micronesia PNG Samoa		12.2 (2895) 77 (158) 24.1 (174) 45 (25)
Aminoglycoside	Gentamicin	PNG	3–37 (37, 61)	45 (25)
Chloramphenicol	Chloramphenicol	PNG	32–96 (37, 61)	
Fluoroquinolone	Ciprofloxacin	Fiji Kiribati Marshall Islands Micronesia PNG Samoa		11.9(2566) 3 (72) 13 (202) 16 (158) 13.3 (526) 13.9 (43)

No studies found for either *K. pneumoniae* or *E. coli* between 2001 and 2010.

**Table 2:** Prevalence of ABR in *Neisseria gonorrhoeae* in PICTs reported by country and date.

*N. gonorrhoeae*				
Antibiotic Class	Antibiotics	PICTs	2000–2010 % or % Range (No. Isolates Tested per Study)	2011–2017 % or % Range (No. Isolates Tested per Study)
Penicillin	Penicillin	Fiji	8.3 (336, 541)	6.7 (252)
		New Caledonia	0.5–47 (197, 208 *, 110)	9 (166)
		PNG	3.6–63 (52, 82, 54)	
Cephalosporin	Ceftriaxone	Fiji		0.4 (541 *)
		Solomon Islands		10 (10)
Fluoroquinolone	Ciprofloxacin	Fiji	0.2–0.6 (541, 336)	
		New Caledonia	1.3–0.5 (79, 197)	6 (166)
		PNG	2 (52)	
Tetracycline	Tetracycline	PNG	19 (52)	

* Intermediate susceptibility.

**Table 3:** Prevalence of ABR in *Shigella* and *Salmonella* spp. (NTS) in the PICTs—reported by country and date.

***Shigella* spp.**				
**Antibiotics**	**PICTs**	**1980–2000** **% (No. Isolates Tested by Study)**	**2001–2010** **% (No. Isolates Tested by Study)**	**2011–2017** **% (No. Isolates Tested by Study)**
Ampicillin	Fiji	79 (205}		
	PNG	86 (94)		91.5 (47)
Amoxicillin	PNG		96 (98)	
Tetracycline	PNG	91(54)		76.6 (47)
Doxycycline	Fiji	63.3 (205)	63.3 (205)	
Tigecycline	PNG			76.6 (47)
Trimethoprim	FIJI	29 (160)		
Co-trimoxazole	PNG		86 (76)	70.2 (47)
Chloramphenicol	Fiji	69.7 (205)		
	Fiji		60 (114)	
	PNG	83 (94)		55.3 (47)
Nalidixic acid	Fiji	3 (37)		
	PNG		15 (13)	
***Salmonella* spp.**				
**Antibiotics**	**PICTs**	**1980–2000** **% (No. Isolates Tested by Study)**	**2001–2010** **% (No. Isolates Tested by Study)**	**2011–2017** **% or % Range (No. Isolates Tested by Study)**
Ampicillin-sulbactam	PNG	53 (38)		8.5 (5)
Amoxicillin-clavulanate	Fr. Polynesia			3.1 (96)
Ceftriaxone	PNG			19 (315)
Doxycycline	Fiji	6 (33)		2.3 (215)
Nalidixic acid	PNG	58 (38)		
Ciprofloxacin	Fiji			0.3 (383)
	PNG			20, 33.3 (5, 15)

**Table 4:** Prevalence of *Staphylococcus aureus* and MRSA * reported in the PICTs by country and date.

*S. aureus* and MRSA *					
Antibiotic Classes	Antibiotics	PICTs	1978–2000 % (No. Isolates Tested per Study)	2001–2010 % or % Range (No. Isolates Tested per Study)	2011–2017 % or % Range (No. Isolates Tested per Study)
Penicillin	Oxacillin	PNG		44 * (9)	6.4–89.4 * (202, 164, 47)
		Samoa		64.7 * (34)	
		Fiji		6.7 * (323)	0.2–2.4 * (437, 2502)
		Kiribati			31 * (36)
		FSM			4 * (113)
		Tonga			17.2 * (430)
	Methicillin	New Caledonia		33.5 *–21 * (202, 544)	
		Samoa		51 * (246)	
		PNG	3 (73)	27.2 * (11)	43.9–81.5 * (164, 47)
		Samoa			24 (389)
	Flucloxacillin	Cook Is			22 (588)
		PNG		36.3 * (11)	
		Samoa		100 * (34)	42 (428)
	Ampicillin	Cook Is			90 (550)
		PNG			93–95.5 * (202, 47)
Tetracycline	Tetracycline	PNG	3–14.8 (66,101)		0.99 * (202)
Lincomycin	Clindamycin	Cook Is			10 (327)
		Samoa			19 (278)
Macrolide	Azithromycin	PNG			46 * (202)
	Erythromycin	Cook Is			17 (557)
		Samoa		29.4 * (34)	33 (312)
Chloramphenicol	Chloramphenicol	Cook Is			5 (259)
		PNG	39.6 (101)	36.3 * (11)	7.4 * (202)
		Samoa			47 (216)
Diaminopyrimidine	Co-trimoxazole	PNG			37.4 * (202)

**Table 5:** Prevalence of ABR in *Streptococcus pneumoniae* in PICTs reported by country and date.

*S. pneumoniae*					
Antibiotic Classes	Antibiotics	PICTs	1970–2000 % or % Range (No. Isolates Tested per Study)	2001–2010 % or % Range (No. Isolates Tested per Study)	2011–2017 % or % Range (No. Isolates Tested per Study)
Penicillins	Penicillin	Fiji		11.4 (195)	
		New Caledonia		21 (544)	
		PICTs		14.4 (298)	
		PNG	12–60.3 (350, 58, 5, 50, 140, 177)	11.7–21.5 (17, 40, 177)	47.9 (121)
	Amoxicillin	New Caledonia		2.7 (544)	
		PICTs		3.7 (298)	
	Amoxicillin/ Clavulanic acid	PICTs		1. 7 (298)	
Cephalosporins	Ceftriaxone	Fiji		0.8 (195)	
		New Caledonia		2.2 (544)	
		PICTs		1.7 (298)	
Tetracyclines	Tetracycline	PNG		4.2–5.8 (176, 17)	
Macrolides	Azithromycin	PNG			4.9 (121)
	Erythromycin	Fiji		1.6 (195)	
Chloramphenicol	Chloramphenicol	PNG	1.5 (121)	2.3–5.8 (176, 17)	0.82 (121)
Diaminopyrimidines	Co-trimoxazole	Fiji		20.3 (195)	
		New Caledonia		2.2 (544)	
		PNG		4–17.6 (176,6,17)	38 (121)

## Supplementary Materials

The following are available online at https://www.mdpi.com/2079-6382/8/1/29/s1, S1: Databases and grey literature sources accessed and search strategies for two databases; S2: Tables (a) and (b) Characteristics describing studies reporting gram-negative and gram-positive pathogens listed by data collection date and ordered by country; S3: Preferred Reporting Items for Systematic reviews and Meta-Analyses extension for Scoping Reviews (PRISMA-ScR).
