# Supplementary material for: Antibiotic Resistance in Pacific Island Countries and Territories: A Systematic Scoping Review
Source: Antibiotics (Basel). 2019 Mar 19;8(1):29. doi: 10.3390/antibiotics8010029 (PMC6466536; doi:10.3390/antibiotics8010029)
Supplement: Supplementary file 1 [file antibiotics-08-00029-s001.zip › antibiotics-462575-supp-1.docx]

**Supplementary material**

**S1.** Databases and grey literature sources accessed and search strategies for two databases

This list includes the electronic databases and grey literature sources accessed for this scoping review

| **Database** | **Access information and Website** |
| --- | --- |
| **Global databases**  - PubMed  - EMBASE  - Web of Science  - Scopus  **Websites browsed for grey literature**  - Google Scholar  - Secretariat of the Pacific Community Public Health Division  - IRIS (Institutional Repository for Information Sharing) World Health Organisation, Western Pacific Region  - Western Pacific Region Index Medicus  - Pacific Health Dialog  Pacific Public Health Surveillance Network publications | Accessed through the Australian National University and University of Queensland Library systems  <https://scholar.google.com/>  <http://www.spc.int/php/>  <http://iris.wpro.who.int/>  <http://www.wprim.org/>  <http://pacifichealthdialog.org.fj/>  <https://www.pphsn.net/> |

Search Strategies for two databases: PubMed and EMBASE

PubMed

Search (1)

((("Drug Resistance, Microbial"[Mesh] OR "Anti-Bacterial Agents"[Mesh] OR "antibiotic resistance"[tw] OR "antimicrobial resistance"[tw] OR antibacterial resistance[tw] OR "multi-drug resistance"[tw] OR "drug resistance"[tw]) OR (("anti-bacterial"[All Fields] AND "agents"[All Fields]) OR ("anti-bacterial agents"[All Fields] OR "antibiotic"[All Fields]))) AND (resistance[All Fields] OR susceptible[All Fields]))) OR ("extended-spectrum beta-lactamase"[tw] OR beta-lactamase[tw] OR esbl[tw] OR gram-negative[tw] OR gram-positive[tw] OR pneumoniae[tw] OR Klebsiella[tw] OR salmonella[tw] OR typhoid[tw] OR Enterobacter[tw] OR "acinetobacter baumannii"[tw] OR "streptococcus pneumoniae"[tw] OR pseudomonas[tw] OR pneumococcus[tw] OR "staphylococcus aureus"[tw] OR "methicillin resistant staphylococcus aureus"[tw] OR MRSA[tw] OR "escherichia coli"[tw] OR shigella[tw] OR cholera[tw] OR cholerae[tw] OR haemophilus[tw] OR "neisseria meningitidis"[tw] OR "neisseria gonorrhoeae"[tw] OR pseudomonas[tw]) AND "humans"[MeSH Terms]

Search (2)

("Pacific Islands"[Mesh] OR "Pacific islands"[tw] OR "Micronesia"[Mesh] OR Micronesia[tw] OR Polynesia[tw] OR Melonesia[tw] "Cook islands"[tw] OR Fiji[tw] OR "Wallis and Futuna"[tw] OR "French Polynesia"[tw] OR "Federated States of Micronesia"[tw] OR Guam[tw] OR Kiribati[tw] OR "Marshall islands"[tw] OR Nauru[tw] OR Niue[tw] OR "New Caledonia"[tw] OR Palau[tw] OR "Pitcairn islands"[tw] OR "Papua New Guinea"[tw] OR PNG[tw] OR Samoa[tw] OR "Solomon islands"[tw] OR Tonga[tw] OR Tuvalu[tw] OR Tokelau[tw] OR Vanuatu[tw]) AND "humans"[MeSH Terms]

Combination of Search (1) AND Search (2)

EMBASE

Search (#1)

('pacific islands'/exp OR 'pacific islands' OR 'micronesia'/exp OR micronesia OR 'melanesia'/exp OR melanesia OR 'polynesia'/exp OR polynesia OR 'cook islands/exp' OR 'cook islands' OR 'fiji'/exp OR fiji OR 'wallis and futuna'/exp OR 'wallis and futuna' OR 'french polynesia'/exp OR 'french polynesia' OR 'federated states of micronesia'/exp OR 'federated states of micronesia' OR 'guam'/exp OR guam OR 'kiribati'/exp OR kiribati OR 'marshall islands'/exp OR 'marshall islands' OR 'nauru'/exp OR nauru OR 'niue'/exp OR niue OR 'new caledonia'/exp OR 'new caledonia' OR 'palau'/exp OR palau OR 'pitcairn islands' OR 'papua new guinea'/exp OR 'papua new guinea' OR 'png' OR 'samoa'/exp OR samoa OR 'solomon islands'/exp OR 'solomon islands' OR 'tonga'/exp OR tonga OR 'tuvalu'/exp OR tuvalu OR 'tokelau'/exp OR tokelau OR vanuatu OR 'vanuatu'/exp) AND [embase]/lim

Search (#2) *

('indonesia'/exp OR indonesia OR 'philippines'/exp OR philippines OR 'timor leste'/exp OR 'timor leste') AND [embase]/lim

Search (#3)

('antibiotic resistance'/exp OR 'antibiotic resistance' OR 'antimicrobial resistance'/exp OR 'antimicrobial resistance' OR 'multi-drug resistance'/exp OR 'multi-drug resistance' OR 'drug resistance'/exp OR 'drug resistance' OR 'anti-bacterial agents'/exp OR 'anti-bacterial agents' OR 'antibacterial'/exp OR 'antibacterial' OR 'disease susceptibility'/exp OR 'disease susceptibility' OR 'susceptibility'/exp OR 'susceptibility' OR 'extended-spectrum beta-lactamase'/exp OR 'extended-spectrum beta-lactamase' OR 'beta lactamase'/exp OR 'beta lactamase' OR 'esbl'/exp OR esbl OR 'gram negative' OR 'gram positive' OR pneumoniae OR 'klebsiella'/exp OR klebsiella OR 'salmonella'/exp OR salmonella OR 'typhoid'/exp OR typhoid OR 'enterobacter'/exp OR enterobacter OR 'acinetobacter baumannii'/exp OR 'acinetobacter baumannii' OR 'streptococcus pneumoniae'/exp OR 'streptococcus pneumoniae' OR 'pneumococcus'/exp OR pneumococcus OR 'staphylococcus aureus'/exp OR 'staphylococcus aureus' OR 'methicillin resistant staphylococcus aureus'/exp OR 'methicillin resistant staphylococcus aureus' OR 'mrsa'/exp OR mrsa OR 'escherichia coli'/exp OR 'escherichia coli' OR 'shigella'/exp OR shigella OR 'cholera'/exp OR cholera OR cholerae OR 'haemophilus'/exp OR haemophilus OR 'neisseria meningitidis'/exp OR 'neisseria meningitidis' OR 'neisseria gonorrhoeae'/exp OR 'neisseria gonorrhoeae' OR 'pseudomonas'/exp OR pseudomonas) AND [embase]/lim

Combine (#1 AND #3) NOT #2

Refined by Sources [EMBASE articles only], Publication types [Article, Review, Article in press, Conference paper]

*The Émbase descriptor, ‘Pacific Islands’ includes Indonesia, Timor Leste and Philippines
