# Supplementary material for: Antibiotic Resistance in Pacific Island Countries and Territories: A Systematic Scoping Review
Source: Antibiotics (Basel). 2019 Mar 19;8(1):29. doi: 10.3390/antibiotics8010029 (PMC6466536; doi:10.3390/antibiotics8010029)
Supplement: Supplementary file 1 [file antibiotics-08-00029-s001.zip › antibiotics-462575-supp-2.docx]

**Supplementary material**

**S2 Table(a). and S2 Table(b). Characteristics describing studies reporting gram-negative and gram-positive pathogens in PICTs**

**Table 1.** Characteristics describing studies reporting gram-negative pathogens listed by data collection date and ordered by country.

| **Ref** | **Country [n]** | **Study date** | **Study design** | **Age** | **Infection** | **Organism** | **Specimen** | **ABR** | **Test** | **Acquired** |
| --- | --- | --- | --- | --- | --- | --- | --- | --- | --- | --- |
| [25] | Cook Is | 2017 | Guideline | All | Bloodstream, meningitis, UTI, other not listed | *K. pneumoniae,*  *E.coli,*  *N. meningitidis,*  *Enterobacter spp, Proteus spp,*  *P. aeruginosa* | Blood, urine, CSF, other not listed | AMC, AML, Meropenem, SXT, GM, CRO, CIP, CXM | Disk diffusion | nr |
| [29] | Fiji [252] | 2013 | Surveillance report | Adult | Gonorrhoea | *N. gonorrhoeae* | Urethral, cervical | P, NSR, CIP | Disk diffusion Agar dilution | Comm |
| [15] | Fiji [418] | 2012 | Retrospective cohort | Neonate | Sepsis, respiratory problems in NICU | *E. coli*  *K. pneumoniae*  *A. baumannii* | Rectal, blood, endotracheal tube, urine, blood | P, GM, AM, CRO, CTX | n/r | Hosp |
| [19] | Fiji [663] | 2011-12 | Retrospective cohort | Adult | BSI, VAPs in ICU | *K. pneumoniae Acinetobacter spp.*  *P. aeruginosa, Enterobacter spp.* | Blood pleural fluid, urine,  wound,  surgical drain | n/r | n/r | Hospital |
| [44] | Fiji [1847] | 2008-12 | Descriptive study | All | Enteric fever | *S. typhi* | Blood, stool, rectal, urine | CIP, CAZ, CTX | Broth microdilution Disk diffusion | Comm |
| [31] | Fiji [336] | 2010 | Surveillance report | Adult | Gonorrhoea | *N. gonorrhoeae* | Urethral, cervical | P, NAR, CIP, TET | Disk diffusion  Agar dilution | Comm |
| [30] | Fiji [541] | 2009 | Surveillance report | Adult | Gonorrhoea | *N. gonorrhoeae* | Urethral, cervical | P, NAR, CIP, TET | Disk diffusion Agar dilution | Comm |
| [28] | Fiji [18] | 2007 | Outbreak report | Neonate | Blood stream | *E. aerogenes* | Blood | AM, P,  SXT, GM, CRO, CET | Disk diffusion | Hospital |
| [47] | Fiji [205;1] | 1996-99  [2005] | Descriptive study; case study | All | Shigellosis | *Shigella spp.*  *S. flexneri* | Stool | AM, DXT, C, NAR, CEC, CET, TMP | n/r | Comm |
| [27] | French Polynesia [1] | 2015 | Case study | Adult | Cholesteatoma | IMI-1 *E. cloacae* | Surgical site | AMC, IMP,  $ see note below | Disk diffusion  E-test gradient diffusion | n/r |
| [43] | French Polynesia [96] | 2008-13 | Laboratory study | n/r | Salmonella (NTS) | *S. enteritidis* | Stool | AML | n/r | Comm |
| [24] | French Polynesia [24] | 2004 | Outbreak study | All | MDR bacteria | *A. baumannii* | Rectal, BAF, urinary and central catheter, tracheal aspirate | IMP, MEM, P, CIP, TET, C, SXT, GM, NET | Disk diffusion E-test gradient diffusion | Hospital |
| [29] | New Caledonia [166] | 2013 | Surveillance report | Adult | Gonorrhoea | *N. gonorrhoeae* | Urethral, cervical | P, NAR, CIP | Disk diffusion  Agar dilation | Comm |
| [17] | New Caledonia [119] | 2008-13 | Retrospective cohort | All | Pneumonia, liver abscess meningitis soft tissue infection bacteraemia | *K. pneumoniae* | Blood | AM. TIC, PIP | Disk diffusion ViteK 2 | Comm |
| [31] | New Caledonia [197] | 2010 | Surveillance report | Adult | Gonorrhoea | *N. gonorrhoeae* | Urethral, cervical | P. NAR, CIP, TET | Disk diffusion  Agar dilation | Comm |
| [30] | New Caledonia [133] | 2009 | Surveillance report | Adult | Gonorrhoea | *N. gonorrhoeae* | Urethral, cervical | P. NAR, CIP | Disk diffusion  Agar dilation | Comm |
| [38] | New Caledonia [110] | 2009 | Laboratory study | Adult | Gonorrhoea | *N. gonorrhoeae* | Urethral, cervical | P | E-test gradient diffusion | Comm |
| [35] | New Caledonia [47] | 2008 | Laboratory study | Adult | Gonorrhoea | *N. gonorrhoeae* | urethral,  cervical | NAR, CRO | E-test gradient diffusion | Comm |
| [36] | New Caledonia [208] | 2005-7 | Laboratory study | All | STIs | *N. gonorrhoeae* | Urethral, cervical | P | Disk diffusion  E-test gradient diffusion | Comm |
| [22] | New Caledonia [202] | 2004 | Case control | Adult | MDR bacteria | *P.aeruginosa*  *E. coli,*  *K. pneumoniae*  *S. maltophilia*  *A. baumannii* | Blood | IMP, MEM, CAZ, CRO, AM/ Sulbactam | Disk diffusion  E-test gradient diffusion | Hospital |
| [23] | New Caledonia [202] | 2004 | Laboratory study | Adult | MDR bacteria | *A. baumannii* *P. aeruginosa* *Enterobacteriaceae*  *S. maltophilia* | Blood | IMP, MEM, CAZ, CRO, AM-Sulbactam | Disk diffusion  E-test gradient diffusion | Hospital |
| [37] | New Caledonia [107] | 2003-4 | Laboratory study | Adult | STIs | *N. gonorrhoeae* | Urethral,  cervical | P | Disk diffusion  E-test gradient diffusion | Comm |
| [21] | PICs: Fiji, Samoa, FSM, PNG | 2012 | Surveillance report | All | n/r | *E. coli*  *K. pneumoniae*  *Salmonella (NTS)*  *N. gonorrhoeae* | n/r | CAZ, CTX, CRO, CIP, CFM, | n/r | [Hospital  Comm] |
| [42] | PICs: Samoa Fiji | 2005-10 | Retrospective review | All | Enteric fever | *S. typhi*  *S. Paratyphi* | Blood, stool, urine | NAR | n/r | Comm |
| [40] | PICs: Fiji Tonga | 1991-1998 | Surveillance report | All | n/r | *Enterobacter spp.*  *E. coli,*  *P. vulgaris,*  *Shigella spp.* | n/r | GM, AM, C, TET | n/r | n/r |
| [41] | PNG [216] | 2010-11 | Surveillance report | n/r | shigellosis,  enteric fever | *Shigella spp.*  *S. typhi* | Stool | AM, TET, CIP, NAR, CTZ, CRO | PCR | Comm |
| [48] | PNG [321] | 2009-11 | Outbreak report | All | Cholera | *V. cholerae* | Stool | AM, C, CIP, E, NAR, CTZ, TET, NOR | Disk diffusion | Hospital  Comm |
| [34] | PNG [197] | 2010 | Surveillance report | Adult | Gonorrhoea | *N. gonorrhoeae* | [Urethra cervical] | P, NAR, CIP, TET | Disk diffusion  E-test gradient diffusion | Comm |
| [12] | PNG [115] | 2008-9 | Prospective cohort | All | Soft tissue injuries, fractures, appendicitis, bites, ulcer, abscess | *K. pneumoniae*  *P. aeruginosa, Alcaligenes spp*. | Blood | TET, C, CTZ, GM, CIP | Disk diffusion  E-test gradient diffusion | Hospital Comm |
| [76] | PNG [22] | 2006-9 | Prospective cohort | Child | Bacterial meningitis | *H. influenzae* | Blood, CSF | P, C, SXT, TET | Disk diffusion  E-test gradient diffusion | Comm |
| [33] | PNG [54] | 2009 | Surveillance report | Adult | Gonorrhoea | *N. gonorrhoeae* | [Urethra cervical] | P, NAR, CIP | Disk diffusion  E-test gradient diffusion | Comm |
| [55] | PNG [854] | 2010-12 | Cross-sectional survey | Adult | Nasopharyngeal bacteria carriage | *H. influenzae* | Nasopharyngeal | P,CRO,OX,CTZ | Disk diffusion E-test gradient diffusion | Comm |
| [46] | PNG [3419] | 2000-9 | Outbreak report | Child | Diarrhoea | *Shigella spp.* | Stool | AM, C, NAR, CTZ | n/r | Comm |
| [16] | PNG [57] | 2007-8 | Outbreak report | Neonate | Bacteraemia | *K. pneumoniae* | Blood | n/r | n/r | Hospital |
| [34] | PNG [210] | 2004-5 | Cross-sectional survey | Adult | STIs | *N. gonorrhoeae* | Urethral cervical | PIP, CIP, TET | Disk diffusion E-test gradient diffusion | Comm |
| [39] | PNG [121] | 2001 | Cross-sectional survey | Child | Meningitis | *H. influenzae N. meningitidis*  *E. coli* | CSF | C | n/r | Comm |
| [61] | PNG [346] | 1997-00 | Clinical trial | Neonate child | Meningitis | *H. influenzae* | CSF | C | Disk diffusion | Comm |
| [13] | PNG [5331] | 1998-00 | Descriptive study | Child | Meningitis | *H. influenzae* | Blood CSF | C | n/r | Hospital  Comm |
| [14] | PNG [61] | 1997-8 | Prospective cohort | Neonate  child | Pneumonia  meningitis diarrhoea septicaemia septic arthritis | *K. pneumoniae*  *P. aeruginosa*  *E. coli*  *Enterobacter spp.* | Blood, CSF,  Abscess,  lung aspirate | C, GM | n/r | Hospital  Comm |
| [20] | PNG [1] | 1991-2 | Case reports | Adult | Pneumonia | *Klebsiella spp*. | Blood  pleural aspirate | GM, P, AM, SM, C, CTX | n/r | Hospital |
| [69] | PNG [917] | 1980-89 | Descriptive study | Neonate  child | Otitis media, pneumonia, meningitis,  nasal discharge | *H. influenzae* | Blood, CSF, nasopharyngeal and nasal | P | ACP (Stokes method) | Comm |
| [66] | PNG [103] | 1985-87 | Descriptive study | All | Nasopharyngeal carriage | *H. influenzae*  other gram-negative species | Nasal | AM, TET, C, GM, CTX | Replica plating method | Comm |
| [18] | PNG [73] | 1987 | Descriptive study | All | Mixed bacterial infections | *Salmonella (NTS),*  *Shigella spp.*  *Klebsiella spp.*  *E. coli Campylobacter spp*. | Blood, CSF, lung aspirates, URT | C, TET, AM, SXT, CTZ | ACP (Stokes method) | Comm |
| [63] | PNG [83] | 1978-81 | Prospective cohort | Child | Pneumonia | *H. influenzae* | Blood  lung and nasopharyngeal aspirates | P | Agar dilution | Comm |
| [26] | Samoa | 2015 | Guideline | All | Bloodstream, meningitis,  UTI, other not listed | *Salmonella typhi*  *K. pneumoniae*  *H. influenzae*  *A. baumannii Enterobacter spp. Proteus spp.*  *P. aeruginosa* | Blood, urine, CSF, other not listed | AM, AML, CIP, GM, SXT, C, CRO, CXM | Disk diffusion | nr |

AM ampicillin, AML amoxicillin, AZ azithromycin, CAR carbapenem, FOX cefoxitin, CTX cefotaxime, CAZ ceftazidime, CXM cefuroxime, CRO ceftriaxone, CET cephalothin, C chloramphenicol, CIP ciprofloxacin, CST colistin, CTZ cotrimoxazole, DXT doxycycline, E erythromycin, GM gentamicin, IMI imipenem, MEM meropenem, MZ metronidazole, OXA oxacillin, NAR nalidixic acid, NIT nitrofurantoin, PIP piperacillin, TET tetracycline, TMP trimethoprim, SXT sulfamethoxazole-trimethoprim, SM streptomycin, VAN vancomycin.

$ *E. cloacae* also resistant to aminopenicillins and carboxypenicillins, first and second generation cephalosporins.

* = Pacific islanders in New Zealand; ** = Pacific islanders in Hawaii; *** Pacific islanders in Australia.

**Table 2.** Characteristics describing studies reporting gram-positive pathogens listed by data collection date and ordered by country

| **Ref** | **Country [n]** | **Study date** | **Study design** | **Age** | **Infections** | **Organisms** | **Specimens** | **ABR** | **Tests** | **Acquired** |
| --- | --- | --- | --- | --- | --- | --- | --- | --- | --- | --- |
| [25] | Cook Is | 2017 | Guideline | All | nr | *S. aureus*  *K. pneumoniae*  *S. pyogenes*  *S. pneumoniae* | Pus, wound, aspirates, sputum, blood, urine | AM, P, AML, CLI, DOX, E, SXT, C, Flucloxacillin, | Disk diffusion | nr |
| [57] | Cook Is [158] | 1958 | Descriptive study | All | Skin and soft tissue,  abscess, ulcer, ears | *S. aureus,*  *Group A Streptococcus,*  *C. diphtheriae* | Nasopharyngeal,  skin and soft tissue | P | Disk diffusion | Comm |
| [15] | Fiji [418] | 2012 | Retrospective cohort | Neonate | Sepsis,  pneumonia | *Streptococcus spp*  CNS | Rectal, blood,  Endotracheal tube | P, AM, CRO, CTX | n/r | Hospital |
| [19] | Fiji [437] | 2011-12 | Retrospective cohort | Adult | Nosocomial infections, BSI, VAPs in ICU | *S. aureus* | Blood, urine,  pleural fluid,  skin and soft tissue, surgical | n/r | n/r | Hospital |
| [53] | Fiji [455] | 2006-7 | Prospective cohort | Child | Impetigo | *S. aureus* | Skin and soft tissue | Methicillin | Broth microdilution Vitek2 | Comm |
| [53] | Fiji [36] | 2006-7 | Descriptive study | All | Bacteraemia, diabetic foot,  surgical site | *S. aureus* | Surgical site, CSF, blood,  skin and soft tissue | Methicillin | Vitek2 | Hospital  Comm |
| [82] | Fiji [62] | 2005-7 | Surveillance report | All | Necrotising fasciitis | *S. pyogenes* | Blood | P, E, C, CLI | Disk diffusion | n/r |
| [71] | Fiji [774] | 2003-4 | Cross-sectional survey | Child | Otitis media,  bacteraemia,  pneumonia | *S. pneumoniae* | Nasopharyngeal | P, CTZ, CRO, E | Disk diffusion,  E-test gradient diffusion | Comm |
| [70] | French Polynesia  [298] | 1999-2001 | Laboratory study | All | Meningitis | *S. pneumoniae* | Blood, CSF | P, AML, CTX | Disk diffusion,  E-test gradient diffusion | n/r |
| [68] | New Caledonia  [1436] | 2000-7 | Outbreak report | All | Pneumonia  meningitis | *S. pneumoniae* | Blood, CSF,  Pleural fluid | P, AML, CTX | Disk diffusion,  E-test gradient diffusion | Comm |
| [78] | New Caledonia  [90] | 2006 | Retrospective cohort | All | Necrotising fasciitis,  bacteraemia, myositis, arthritis,  pneumonia, erysipelas | *S. pyogenes* | Skin and soft tissue,  blood, synovial, pleural,  amniotic and spinal fluid | TET | Disk diffusion | Comm |
| [22] | New Caledonia  [202] | 2004 | Case control | n/r | MDR bacteria | *S. aureus* | n/r | Methicillin | Disk diffusion,  E-test gradient diffusion | Hospital |
| [60] | New Caledonia  [544] | 2002-3 | Cross-sectional survey | Neonate | Meningitis | *S. pneumoniae* | Nasopharyngeal | P, CTX, AML | Disk diffusion,  E-test gradient diffusion | Comm |
| [81] | New Caledonia  [1] | 2001 | Case report | Adult | Nosocomial | *VRE* | Stool | VAN | Disk diffusion,  E-test gradient diffusion, PCR | Hospital |
| [70] | New Caledonia  [298] | 1999-2001 | Laboratory study | All | Meningitis | *S. pneumoniae* | Blood, CSF | P, CTX, AM | ATB PNO strip,  E-test gradient diffusion | n/r |
| [21] | PICTs:  Fiji  FSM  PNG  Samoa | 2014 | Surveillance report | All | n/r | *All gram-positive bacteria* | n/r | CAZ, CTX, CRO, CIP, CEF, OXA, P | n/r | n/r |
| [82] | PICTs  [43]  Fiji, Kiribati,  Solomon Is | 2011 | Cross-sectional survey - questionnaire | n/r | n/r | *n/r* | n/r | n/r | n/r | n/r |
| [59] | PICTs***  [5000]  Samoa  Tonga | 1993-2004 | Retrospective cohort | Neonate  child | Sepsis,  skin and soft tissue,  septic arthritis, pneumonia, osteomyelitis, abscess,  limb trauma | *S. aureus* | Blood | Methicillin, E, Flucloxacillin | n/r | Hospital  Comm |
| [56] | PICTs**  [1389]  Pacific Islanders | 2001-3 | Descriptive study | All | Pneumonia, ulcer, abscess, wounds, impetigo, cellulitis | *S. aureus* | Skin and soft tissue | Methicillin | n/r | Comm |
| [52] | PICTs*  [139]  Samoa  Tonga | 1998-1990 | Prospective cohort | All | Cellulitis, wounds, bacteraemia, pneumonia, ulcers, abscess | *S. aureus*  *MRSA* | Wound | P, CIP, E, GM, TET, SXT, Methicillin, | VITEK GPS, PCR | Comm |
| [40] | PICTs:  Fiji  Tonga | 2002 | Surveillance  report | All | n/r | All gram-positive bacteria | n/r | AM, GM, CTX, SXT, TET, C, OXA | n/r | n/r |
| [49] | PNG  [70] | 2012-17 | Prospective | Child | Osteomyelitis | *S. aureus*  *Enterobacteriaceae* | Aspirate, blood, pus, bone debris | GM, P, AM, CRO Methicillin, OX | Disk diffusion | Comm |
| [55] | PNG  [854] | 2009-12 | Randomised controlled trial | All | Nasopharyngeal bacteria carriage | *S. aureus* | Nasopharyngeal | P, CRO, OX, CTZ | Disk diffusion  E-test  Gradient diffusion | Comm |
| [12] | PNG  [115] | 2008-9 | Observational prospective study | All | Blood stream,  skin and soft tissue,  wound, abscess | *S. aureus*  *S. pneumoniae* | Blood | Methicillin, P, OX, Flucloxacillin | Disk diffusion  E-test gradient diffusion | Comm |
| [76] | PNG  [22] | 2006-9 | Laboratory study | Child | Bacterial meningitis | *S. pneumoniae* | Blood, CSF | P, C, SXT, TET | Disk diffusion  E-test gradient diffusion | Comm |
| [75] | PNG  [51] | 1998-2008 | Observational descriptive study | Adult | Bacterial meningitis | *Streptococcus spp.* | CSF | E, CEC, TET, P, C, AM, CRO, AML, CTX, GM | n/r | n/r |
| [65] | PNG  [1884] | 1996-2005 | Observational descriptive | n/r | Bacterial meningitis | *S. pneumoniae* | CSF | P, CZ, TET, C, OX, AM | Disk diffusion  E-test gradient diffusion | Comm |
| [39] | PNG  [121] | 2001 | Observational descriptive | Child | Meningitis | *S. pneumoniae* | CSF | C | n/r | Comm |
| [61] | PNG  [346] | 1997-00 | Clinical trial | Neonate  child | Meningitis | *S. pneumoniae* | CSF | C | Disk diffusion | Comm |
| [14] | PNG  [61] | 1997-8 | Observational descriptive | Neonate  child | Pneumonia, meningitis, diarrhoeal disease, septicaemia,  septic arthritis | *Enterobacter spp.* | Blood, CSF, lung aspirates,  abscess | C, GM | n/r | Hospital  Comm |
| [69] | PNG  [917] | 1980-1989 | Observational descriptive |  | Otitis media, pneumonia, meningitis,  nasal discharge | *S. pneumoniae* | Blood, CSF, nasopharyngeal | P | ACP (Stokes method) | Comm |
| [51] | PNG  [1] | 1989 | Case report | Adult | Pneumonia | *S. aureus* | CSF | Methicillin | n/r | Comm |
| [66] | PNG  [103] | 1985-87 | Retrospective  cohort | All | Nasopharyngeal bacteria carriage | *S. pneumoniae* | Nasopharyngeal wound | TET, GM, C, AM, CTX | Replica plating method | Comm |
| [54] | PNG  [101] | 1977-1987 | Retrospective  cohort | All | Skin and soft tissue, pneumonia, arthritis, abscess, endocarditis | *S. aureus* | Blood, wound, sputum, pleural and synovial fluid | P, TET, C, SM | Disk diffusion | Hospital  Comm |
| [18] | PNG  [73] | 1987 | Descriptive  study | All | Skin and soft tissue | *S. aureus* | Wound | P, Methicillin, E, C, TET, GM, SXT | ACP (Stokes method) | Comm |
| [58] | PNG  [480] | 1982-3 | Cross-sectional survey | Neonates  child | Skin and soft tissue,  ulcers, scabies | *S. aureus*  *S. pneumoniae* | Wound, pus | Methicillin, P, E, C, TET | Disk diffusion | Comm |
| [63] | PNG  [83] | 1978-81 | Prospective  study | Child | Pneumonia | *S. pneumoniae* | Blood, lung, nasopharyngeal | P | Agar dilution | Comm |
| [64] | PNG  [58] | 1978 | Retrospective  study | All | Meningitis. bacteraemic pneumonia, bacteraemia | *S. pneumoniae* | Blood, CSF | P | Disk diffusion, Plate titration | Comm |
| [73] | PNG  [530] | 1967-68 | Descriptive  study | All | Pneumonia, meningitis | *Pneumococcus* | URT | P, Methicillin, CET, CER | Disk diffusion, Plate titration | Comm |
| [26] | Samoa | 2017 | Guideline | All | nr | *S. aureus*  *K. pneumoniae*  *S. pyogenes*  *S. pneumoniae* | Pus, wound, aspirates, sputum, blood, urine | AM, P, AML, C, CLI, DOX, E, SXT, Flucloxacillin, | Disk diffusion | nr |
| [50] | Samoa  [388] | 2007-8 | Cross-sectional survey | All | Skin and soft tissue,  abscess | *S. aureus* | Wound | Methicillin | Disk diffusion, Broth microdilution | Comm |
| [80] | Solomon Is [24] | 1968-90 | Laboratory study | n/r | n/r | *E. faecalis* | Stool | P, AM, VAN | Broth microdilution | n/r |
| [70] | Wallis Futuna  [298] | 1999-01 | Laboratory  study | All | Meningitis | *S. pneumoniae* | Blood, CSF | P, CTX, AML | ATB PNO  strip | n/r |

AM ampicillin, AML amoxicillin, AZ azithromycin, CAR carbapenem, FOX cefoxitin, CTX cefotaxime, CAZ ceftazidime, CXM cefuroxime, CRO ceftriaxone, CET cephalothin, C chloramphenicol, CIP ciprofloxacin, CST colistin, CTZ cotrimoxazole, DXT doxycycline, E erythromycin, GM gentamicin, IMI imipenem, MEM meropenem, MZ metronidazole, OXA oxacillin, NAR nalidixic acid, NIT nitrofurantoin, PIP piperacillin, TET tetracycline, TMP trimethoprim, SXT sulfamethoxazole-trimethoprim, SM streptomycin, VAN vancomycin.

$ *E. cloacae* also resistant to aminopenicillins and carboxypenicillins, first and second generation cephalosporins.

* = Pacific islanders in New Zealand; ** = Pacific islanders in Hawaii; *** Pacific islanders in Australia.
